# Does the Finger-to-Nose Test measure upper limb coordination in chronic stroke?

**DOI:** 10.1186/s12984-016-0213-y

**Published:** 2017-01-23

**Authors:** Marcos R. M. Rodrigues, Matthew Slimovitch, Gevorg Chilingaryan, Mindy F. Levin

**Affiliations:** 1 School of Physical and Occupational Therapy, McGill University, 3654 Prom Sir-William-Osler, Montréal, QC H3G 1Y5 Canada; 2 Feil and Oberfeld Research Center, Jewish Rehabilitation Hospital, site of Center for Interdisciplinary Research in Rehabilitation of Greater Montreal (CRIR), Laval, Canada; 3 Faculty of Medicine, McGill University, Montreal, Canada

**Keywords:** CVA (cerebrovascular accident), Motor skills disorders, Upper extremity, Outcomes assessment

## Abstract

**Background:**

We aimed to kinematically validate that the time to perform the Finger-to-Nose Test (FNT) assesses coordination by determining its construct, convergent and discriminant validity.

**Methods:**

Experimental, criterion standard study. Both clinical and experimental evaluations were done at a research facility in a rehabilitation hospital. Forty individuals (20 individuals with chronic stroke and 20 healthy, age- and gender-matched individuals) participated.. Both groups performed two blocks of 10 to-and-fro pointing movements (non-dominant/affected arm) between a sagittal target and the nose (ReachIn, ReachOut) at a self-paced speed. Time to perform the test was the main outcome. Kinematics (Optotrak, 100Hz) and clinical impairment/activity levels were evaluated. Spatiotemporal coordination was assessed with slope (IJC) and cross-correlation (LAG) between elbow and shoulder movements.

**Results:**

Compared to controls, individuals with stroke (Fugl-Meyer Assessment, FMA-UE: 51.9 ± 13.2; Box & Blocks, BBT: 72.1 ± 26.9%) made more curved endpoint trajectories using less shoulder horizontal-abduction. For construct validity, shoulder range (β = 0.127), LAG (β = 0.855) and IJC (β = −0.191) explained 82% of FNT-time variance for ReachIn and LAG (β = 0.971) explained 94% for ReachOut in patients with stroke. In contrast, only LAG explained 62% (β = 0.790) and 79% (β = 0.889) of variance for ReachIn and ReachOut respectively in controls. For convergent validity, FNT-time correlated with FMA-UE (*r* = −0.67, *p* < 0.01), FMA-Arm (*r* = −0.60, *p* = 0.005), biceps spasticity (*r* = 0.39, *p* < 0.05) and BBT (*r* = −0.56, *p* < 0.01). A cut-off time of 10.6 s discriminated between mild and moderate-to-severe impairment (discriminant validity). Each additional second represented 42% odds increase of greater impairment.

**Conclusions:**

For this version of the FNT, the time to perform the test showed construct, convergent and discriminant validity to measure UL coordination in stroke.

## Background

Upper-limb (UL) coordination deficits are commonly observed in neurological patients (e.g., cerebellar ataxia, stroke, etc.). In healthy subjects, goal-directed movement requires synchronized interaction (coordination) between multiple effectors [1–3]. Characterizing UL coordination, however, is challenging for clinicians and researchers because of lack of consensus regarding its definition (e.g., see [4–7]). Nevertheless, definitions usually describe coordinated movement as involving specific patterns of temporal (timing between joints) and spatial (joint movement pattern) variability [1, 2, 8]. However, trajectory formation differs for reaches made in a body-centered frame of reference (egocentric) compared to those relying on mapping of extrinsic space and visuo-motor transformations [9, 10] made away from the body (exocentric). Thus, coordination can be defined as the skill of adjusting temporal and spatial aspects of joint rotations according to the task [11].

Damage to descending pathways due to stroke can lead to movement deficits defined at two levels. At the end-effector level (e.g. hand), variables describe movement performance (time, straightness, smoothness, precision), whereas at the interjoint level, variables describe movement quality (joint ranges of motion, interjoint coordination) [12]. These variables may be affected differently for egocentric and exocentric movements.

Although it is widely recognized that training can improve performance of functional tasks even years after a stroke [13], a valid tool for the measurement of coordination has not yet been established. In healthy individuals, coordinated movements are described in terms of spatial variables, related to the positions of different joints or body segments in space and/or temporal variables, related to the timing between movements of joints/segments during the task [1]. Consideration of task specificity is important in characterizing coordination. In addition, movement may be affected by abnormal stereotypical UL movement synergies and concomitant reduction in kinematic redundancy [10, 14] as well as deficits reducing both movement performance and quality [15, 16].

In clinical practice, coordination is assumed to be measured by the time to perform alternating movements with different end effectors (e.g., supination/pronation of the forearm, sliding the heel up and down the anterior aspect of the shin). Another task commonly used to assess coordination is the Finger-to-Nose test (FNT) [17, 18]. In the standard neurological exam [19], the individual alternately touches their nose and the evaluator’s stationary or moving finger while lying supine, sitting or standing. In the Fugl-Meyer UL Assessment (FMA-UL) [18], the FNT is objectively measured as the difference in time to alternately touch the knee and nose five times between the more- and less-affected arm on a 0 to 2 point scale. Aside from FNT-time, two other features of endpoint performance, arm trajectory straightness/smoothness (tremor) and precision (dysmetria), are estimated qualitatively [18] for a total of six points.

However, the construct validity of FNT-time as an UL coordination measure in individuals with stroke has not been established using detailed kinematic assessment, where construct validity is defined as the degree to which experimentally-determined and theoretical definitions match [20]. For clinicians to use FNT as part of the UL assessment, this assumption must be verified along with its convergent and discriminant validity.

The study objectives were to determine construct, convergent and discriminant validity of FNT-time to measure UL coordination in individuals with chronic stroke using kinematic analysis. We characterized movement parameters during performance of FNT between healthy and stroke subjects. We also related FNT outcomes (time, trajectory straightness, precision) to UL impairment severity and activity limitations. We hypothesized that FNT-time would 1) be related to interjoint coordination measures (construct validity); 2) be correlated with other measures of UL impairment and/or activity limitations (convergent validity); and 3) discriminate between levels of UL impairment (discriminant validity). Preliminary data have appeared in abstract form [21].

## Methods

Forty subjects, 20 healthy controls (9 males, aged 61.7 ± 8.7 years) and 20 with stroke (11 males, aged 61.4 ± 14.6 years) participated (Table 1). Individuals with stroke had unilateral ischemic or hemorrhagic strokes in either hemisphere, 6–192 months previously (mean 50.9 ± 42.2 months) and could perform the test (3–7 on Chedoke-McMaster Arm Scale, CM) [22]. They were excluded if they had unilateral neglect, apraxia or ataxia (visually screened for dysmetria using a pointing task) measured by standard clinical assessment. Individuals in both groups were excluded if they had arm pain, uncorrected vision and/or other neurological or musculoskeletal problems affecting UL movement determined by chart review and/or medical consultation.

**Table 1:** Demographic and clinical data for participants including mean age and SD for both groups, sorted by level of upper-limb impairment (Fugl-Meyer Assessment, FMA-UL) in ascending order

S	Age (yr)/Gender	Affected side R/L D/ND	Time since stroke (mo)	Chedoke (arm/hand) (7/7)	FMA-UE (/66 pts)	FMA-Arm (/42 pts)	CSI Biceps (/16 pts)	CSI Triceps (/16 pts)	FMA Proprio-ception (/8)	BBT (% A/LA)	Lesion Type (I/H), location
1	45/M	R/D	25	3/3	30	20	13	13	5	53	I, fronto-parietal, subcortical
2	72/F	R/D	90	3/3	36	22	10	11	8	45	I, MCA
3	77/M	L/ND	19	4/6	38	16	12	12	2	44	H, BG
4	51/M	L/ND	32	4/4	38	24	7	6	5	23	I, MCA
5	37/F	R/D	192	3/3	40	27	13	13	8	13	I, BG, caudate, IC, posteror temporal, lentiform
6	69/M	R/D	40	4/3	46	29	7	5	7	60	I, corona radiata, caudate, centre semi-ovale
7	55/M	L/ND	31	4/5	49	25	8	5	7	53	I, MCA
8	82/M	R/D	59	5/6	57	18	4	4	7	100	L MCA, temporal R tempo-parietal, lacunar, corona radiata
9	72/M	R/D	66	7/6	57	32	3	3	8	100	I, lacunar, coronal radiata
10	43/F	R/D	13	6/7	58	33	7	4	8	100	I, pons, paramedian
11	66/F	L/ND	69	4/6	58	30	8	6	8	62	H, MCA
12	66/M	L/ND	75	6/7	60	31	4	4	6	78	coronal radiata
13	78/F	R/D	38	6/7	61	33	5	4	8	88	n/i, MCA
14	79/M	R/D	50	7/5	62	36	4	4	6	95	n/i, brainstem
15	64/F	L/ND	42	5/6	62	33	4	5	8	76	I, MCA fronto-parieto-temporal, GP, putamen, IC, caudate
16	78/F	L/D	10	6/7	62	32	4	4	8	96	I, lacunar
17	41/F	L/ND	6	6/6	63	36	4	8	7	100	I, occipital
18	63/F	R/D	74	7/5	64	36	4	4	4	100	intraventicular H, BG
19	64/M	L/ND	6	5/6	64	36	8	5	8	84	n/i, brainstem
20	44/M	L/ND	12	7/7	65	36	4	4	3	72	H, fronto-parietal-temporal
Mean (SD)											
	61.4 (14.6)		50.9 (42.2)	5.0 (1.4)/5.3 (1.5)	51.9 (13.2)	29.3 (6.4)	6.7 (3.2)	6.2 (3.3)	6.3 (2.2)	72.1 (26.9)	
Healthy (*n* = 20)											
	61.7 (8.7)										

*Abbreviations*: *A* Affected side, *BBT* Box and Blocks Test, *BG* Basal Ganglia, *CSI* Composite Spasticity Index, *D* Dominant, *F* Female, *GB* Globus Pallidus, *H* Hemorrhagic, *I* Ischemic, *IC* Internal Capsule, *LA* Less-affected side, *L* Left, *M* Male, *MCA* Middle Cerebral Artery, *ND* Non-dominant, *n/i* no information, *R* Right, *S* Subject

### Ethics, consent and permissions

All participants signed consent forms approved by the Ethics Committee of the Centre for Interdisciplinary Research in Rehabilitation of Greater Montreal (CRIR).

**Fig. 1:**
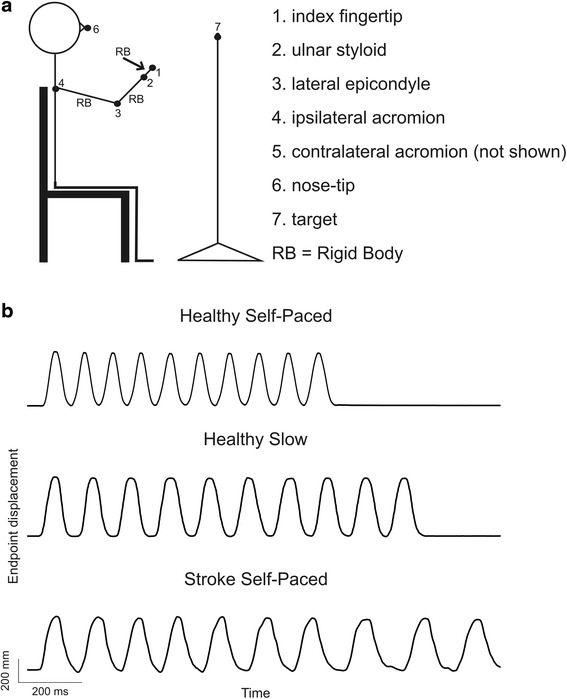
**a** Experimental set up illustrating marker placement and examples of endpoint displacement for finger-to-nose test. Subject sat with one arm partially extended, index finger fully extended and target placed at 90% arm-length at eye-level. The task was to touch the target and then the nose accurately 10 times at a self-paced speed; **b** Examples of 10 trials of endpoint (tip of index finger) displacement over time. First row–healthy subject moving endpoint at self-paced speed; Second row–healthy subject moving endpoint at a slower speed and Third row–Stroke subject moving endpoint a self-paced speed

Participants underwent a 1.5 h clinical evaluation (stroke) and a 2 h experimental session (stroke, controls). The clinical evaluation was performed by a clinician using valid and reliable scales. UL impairment was assessed with the FMA-UL [18] on a 66-point scale, FMA-Arm on a 42-point scale and biceps and triceps spasticity was assessed using the 16-point Composite Spasticity Index (CSI) [23] where 0–9, 10–12 and 13–16 points represent mild, moderate and severe spasticity respectively. UL activity was assessed with the Box and Blocks Test (BBT) [24] and expressed as the percentage of blocks moved by the more-affected compared to the less-affected arm in 60s.

### Experimental task

Subjects performed FNT while comfortably sitting with hips and knees flexed to 90° (Fig. 1a). At a computer-generated tone, subjects alternately touched their nose and a target (2.5 cm diameter circle) with the fingertip. The target was located at nose height and normalized to a distance of 90% arm-length measured from the medial axillary border to the index fingertip to eliminate bias due to inter-subject arm-length differences. Self-paced movements using the subject’s preferred strategy were performed with eyes open. Subjects were instructed to perform continuous movement regardless of corrections even if the target was missed. Discontinuous movement trials were repeated. Two blocks of 10 alternating movements starting with the fingertip on the target were performed in a randomized order for each arm. Since arm movements of the stroke group were slower than controls, two extra blocks of slower movements per arm were done in controls for matched-speed comparisons. No metronome or other timing device was used to indicate movement speed to avoid changes in behavior [25].

### Data collection

Data were recorded from seven markers placed on the index fingertip, ulnar styloid, elbow lateral epicondyle, acromions, nose-tip and target. Three rigid-bodies consisting of six markers each were also placed on the hand dorsum, mid-forearm and mid-arm (Fig. 1a). Data were recorded with a 2-Certus bar Optotrak Motion Analysis System (Northern Digital, Waterloo, ON) for 30s per trial at a sampling rate of 100Hz.

### Data analysis

Because movement direction can be affected by abnormal UL synergies in post-stroke individuals, we analyzed data for each direction separately. Each trial was divided into two segments, yielding 10 target-to-nose egocentric (ReachIn) and 10 nose-to-target exocentric movements (ReachOut). To avoid learning effects and ensure assessment of stable behavior, the first three trials of each block were not considered. Thus, mean values were computed for 14 trials in each direction. Raw x, y, z data were interpolated and smoothed (10 Hz low-pass Weiner filter). Movement onset and offset were determined from the endpoint tangential velocity as points at which the signal rose/fell and remained above/below 10% peak velocity.

Analysis was done at motor performance and quality levels for movements made at matched speeds in each direction. Kinematic measures were those that previously demonstrated moderate to excellent test-retest reliability for midline pointing movement (ICC ≥ 0.6) [26]. Endpoint performance variables were total movement time, trajectory straightness and precision. Movement quality variables were those related to joint rotations and interjoint coordination. For endpoint performance, total movement time was measured from the first target-to-nose segment onset to last nose-to-target segment offset. Movement times for each direction (ReachIn/Out) were defined as the times the fingertip moved from target-to-nose and nose-to-target respectively. Trajectory straightness was defined as the index of curvature (IC), the ratio between the actual endpoint movement path to the shortest distance between the two targets, where IC of one equals a straight-line trajectory. Endpoint precision was computed using the root-mean squared error (RMSE) defined as the difference between the final ReachIn/Out phase endpoint and target/nose x, y, z positions. Movement quality variables were computed as the difference between starting and final joint angles measured in degrees. Elbow flexion/extension (Elbow) was calculated from mid-forearm and mid-arm rigid bodies, where 180° corresponded to the fully extended arm, Shoulder horizontal abduction/adduction (Sh-H-Abd/Add) was calculated from vectors formed between acromial markers and between ipsilateral acromial and lateral epicondyle markers where 0° corresponded to full 90° shoulder abduction. Shoulder flexion (Sh-Flex) was calculated using vectors formed between markers on the ipsilateral acromion and lateral epicondyle and the vertical line through the acromion marker, where 0° indicated the arm alongside the body. Trunk pitch angle was computed as the antero-posterior deviation of the trunk from a vertical line through the midpoint between acromial markers.

Interjoint coordination was assessed with one temporal (LAG) and one spatial (interjoint coordination, IJC) variable. LAG referred to the temporal delay between peak values of Sh-H-Abd and elbow flexion for ReachIn as well as of Sh-H-Add and elbow extension for ReachOut, where 0 ms indicated perfect temporal coincidence. IJC was the slope of the angle-angle plot between shoulder and elbow movements as defined above, where values closer to zero indicated more shoulder compared to elbow movement and values >1 indicated the opposite. Data analysis was done using Matlab v.6.5.1 software (Massachusetts, USA).

### Statistical analysis

For Hypothesis 1, construct validity was assessed using multiple step-wise regression analysis to identify contributions of different kinematic variables to FNT-time (dependent variable), with *p* values of <0.05 and >0.1 used for variable inclusion/rejection, respectively. To identify kinematic variables to include in the model, mean endpoint performance variables (movement time, straightness and precision) and movement quality variables (joint ranges, trunk displacement, LAG, IJC) were compared with 3-factor two-way ANOVAs (group: stroke, healthy; arm: affected, non-affected/dominant, non-dominant; movement direction: ReachIn, ReachOut) and appropriate post-hoc tests with Bonferonni corrections. Normality of distributions and homogeneity of variances were verified with Shapiro-Wilks and Levene’s tests respectively.

For Hypothesis 2, convergent validity was determined by correlating kinematic measures of coordination and FNT-time with clinical measures of UL impairment (FMA-UL, FMA-Arm) and activity limitation (BBT) using Pearson correlations.

For Hypothesis 3, logistic regression analysis was done to estimate discriminant validity of FNT-time (predictor) against FMA-UL scores (dependent variable). For this analysis, FMA-UL and not FMA-Arm scores were used since FMA-UL has established cut-off points to distinguish between levels of severity [27]. Level of severity of hemiparesis was dichotomized into mild and moderate-to-severe based on an FMA-UL cut-off score of 50/66 [27, 28]. Receiver Operating Characteristic (ROC) analysis and Sensitivity/Specificity decision plots [29] identified the optimal cut-off value of FNT-time. In addition, the effect of lesion type (ischemic/hemorrhagic) and site (cortical/subcortical) on FNT-time was determined with Chi-Square tests. All statistical analyses were performed using SPSS Statistics v.20 for Windows (IBM, Armonk, NY) with significance *p* < 0.05.

## Results

### Movements in controls

**Fig. 2:**
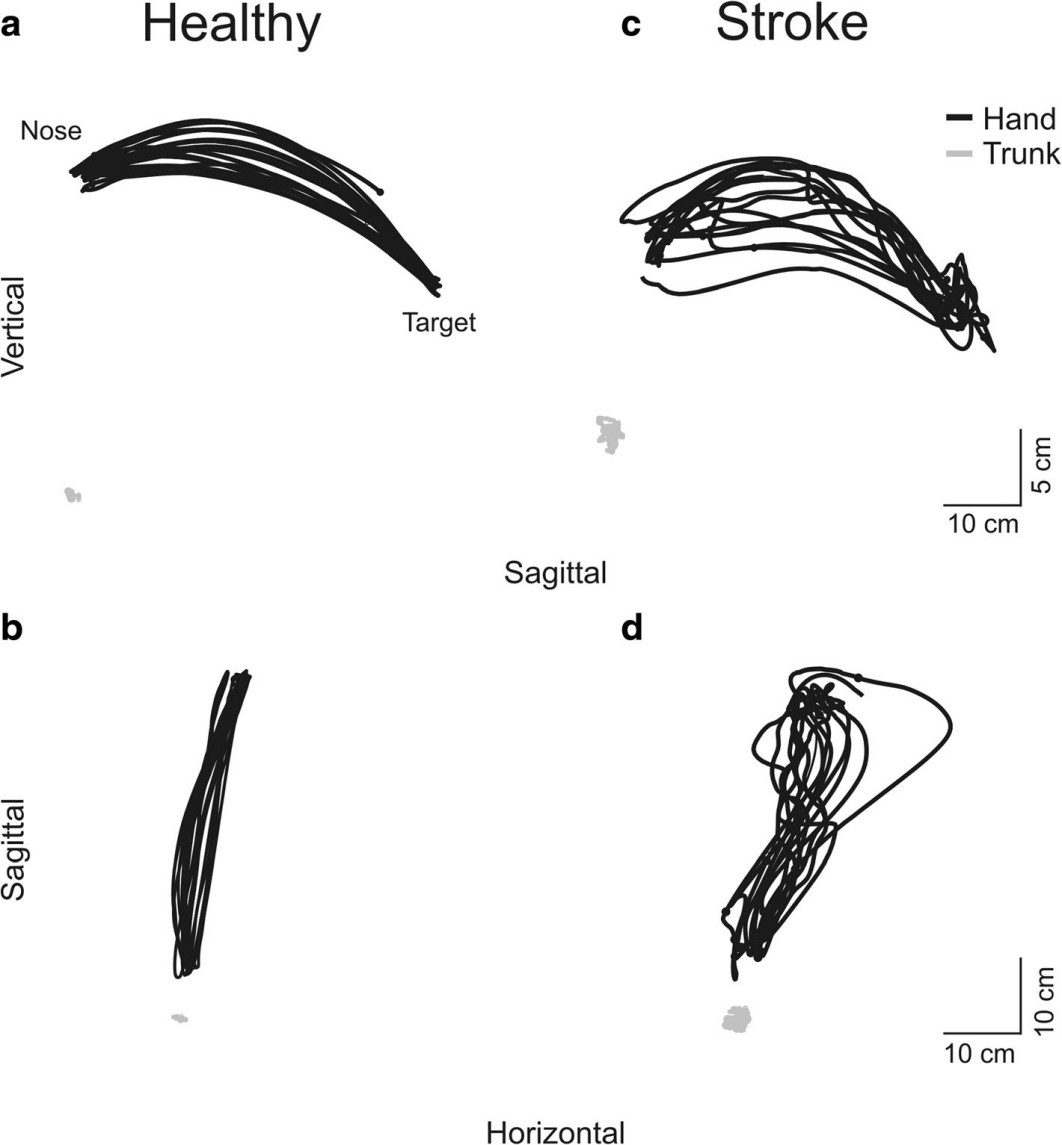
Examples of sagittal (**a**, **c**) and horizontal (**b**, **d**) endpoint (*black lines*) and trunk (*grey lines*) trajectories of 10 trials of the finger-to-nose test in one healthy subject and one subject with stroke

**Fig. 3:**
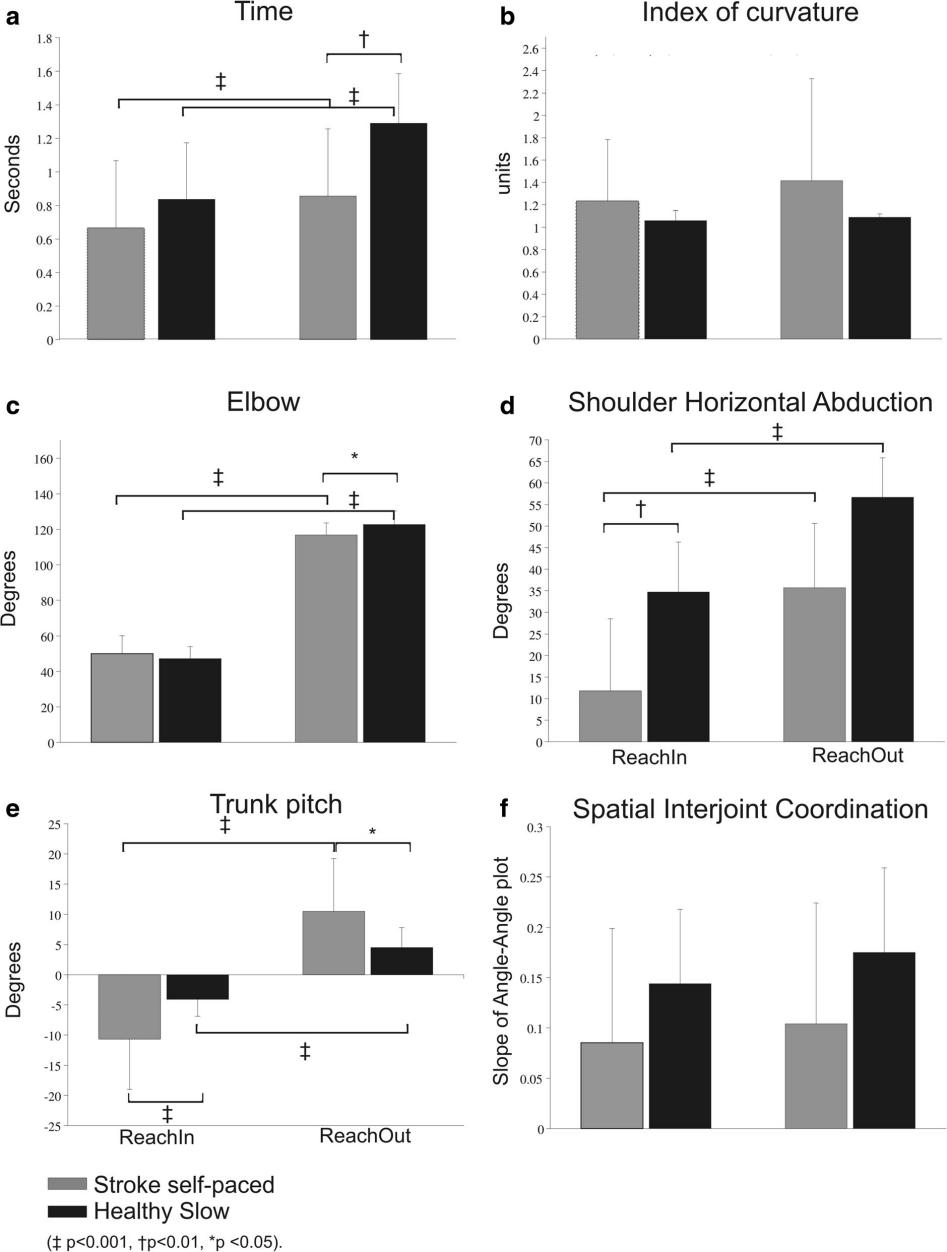
Histograms of main outcome variables; **a** Time to perform the task; **b** Index of curvature; **c** Elbow range of motion; **d** Shoulder horizontal abduction range of motion; **e** Trunk pitch; **f** Spatial interjoint coordination. *Black/grey bars* show means and standard deviations for healthy/stroke groups respectively

Controls made rhythmical endpoint movements with each arm (Fig. 1b). Movements were slightly curved (IC = 1.01–1.09; Fig. 2a, b) and precise (RMSE = 13.2–20.7 mm, ReachIn mean 18.9 ± 5.8 mm, ReachOut mean 17.1 ± 2.9 mm). Error, ranges of Elbow, Sh-H-Abd, Sh-Flex (not shown) and Trunk movement varied with movement direction (Fig. 3a–e). Variables were not affected by learning as evidenced by lack of differences in endpoint and joint variables for either direction between the first and last five movements.

### Movements in individuals with stroke

Clinical UL impairment ranged from moderate to mild (FMA-UL: 30–65, mean 51.9 ± 13.2pts; FMA-Arm: 16–36, mean 29.3 ± 6.4pts; biceps spasticity 3–13pts, mean 6.7 ± 3.2pts; triceps spasticity 3–13pts, mean 6.2 ± 3.3pts) and activity levels varied (BBT: 13–100%, mean 72.1 ± 26.9%; Table 1). Difficulties in reaching with the affected arm were evident in all individuals with stroke (Fig. 2c, d). As expected, similar to controls, index of curvature, Elbow, Sh-H-Abd, Sh-Flex (not shown) and Trunk ranges differed with movement direction (Fig. 3a–e). RMSE errors ranged from 8.0 to 48.6 mm (mean 18.3 ± 10.9 mm) for ReachIn and from 12.5 to 48.6 mm (mean 17.9 ± 8.8 mm) for ReachOut. The stroke group took longer to make outward (ReachOut) compared to inward reaches (ReachIn; *p* < 0.001, Fig. 3a). They also used more elbow extension (F_1,116_ = 22.326, *p* < 0.001, Fig. 3c), Sh-H-Abd (F_1,116_ = 55.181, *p* < 0.001, Fig. 3d) and trunk forward displacement (*p* < 0.001, Fig. 3e) for ReachOut compared to ReachIn movements (F_1,116_ = 144.058, *p* < 0.004). Compared to the less-affected side, FNT-time for the more-affected arm was significantly longer for both ReachIn (*p* = 0.008) and ReachOut (*p* = 0.013) directions. Similar to controls, movement variables were not affected by learning. There were no significant effects of lesion type or location on FNT-time.

### Movements in individuals with stroke compared to controls

Compared to controls, stroke subjects used less Sh-H-Abd for movements in both directions (F_1,114_ = 18.397, *p* < 0.001, Fig. 3d). There were interaction effects between group and movement direction. Individuals with stroke used less elbow extension (F_1,114_ = 4.128, *p* < 0.05, Fig. 3c) and more trunk forward displacement (F_1,116_ = 15.466, *p* < 0.001, Fig. 3e) compared to controls for ReachOut. For ReachIn, individuals with stroke used less Sh-H-Abd compared to controls (Fig. 3d, F_1,114_ = 55.181, *p* < 0.001) and more backward trunk displacement (F_1,116_ = 15.466, *p* < 0.001, Fig. 3e). Errors were similar for both directions and both groups.

### Construct validity

In controls, none of the kinematic variables contributed to FNT-time variance at matched speed (slow) but at faster speeds, LAG explained 62% (β = 0.790) and 79% (β = 0.889) of the variance for ReachIn and ReachOut respectively. In stroke, Sh-H-Abd range (β = 0.127), LAG (β = 0.855) and IJC (β = −0.191) explained 82% of the variance for ReachIn, and LAG (β = 0.971) explained 94% for ReachOut.

### Convergent validity

**Fig. 4:**
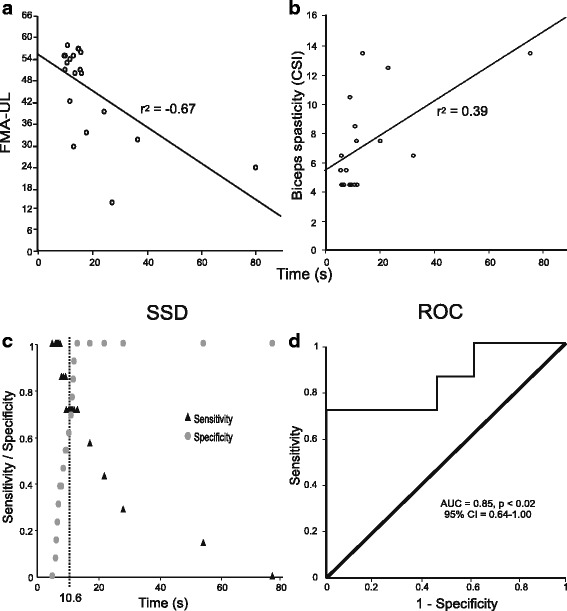
Convergent validity showing correlations between Finger-to-Nose Test (FNT) time and clinical variables (**a**, **b**) and results of discriminant validity analyses (**c**, **d**). Correlations between FNT-time and clinical impairment scores (**a**, Fugl-Meyer Assessment of the Upper Limb (FMA-UL); **b**, biceps spasticity score). **c** Sensitivity/Specificity decision (SSD) plot for time to perform the FNT. Sensitivity (*triangles*) and specificity (*circles*) values were plotted against total time to perform the FNT in seconds. The intersection of both curves (*vertical dotted line*) represents the cut-off time to perform the test (10.6 s) that discriminates between mild and moderate impairment. **d** Receiver Operating Characteristic (ROC) Curve illustrating the area under the curve (AUC), significance level (p value) and 95% confidence interval (95% CI). Diagonal line indicates a 50/50 ratio between sensitivity and specificity of the FNT-time measure

In stroke, FNT-time (10 repetitions) was correlated with impairment severity (FMA-UL: *r* = −0.67, *p* < 0.01, Fig. 4a; FMA-Arm: *r* = −0.60, *p* = 0.005; biceps spasticity: *r* = 0.39, *p* < 0.05, Fig. 4b) and activity level (BBT: *r* = −0.56, *p* < 0.01) but not with proprioception. There was a tendency for a positive relationship between time and LAG (*r* = 0.46, *p* = 0.055) in stroke.

Another aspect of FNT, clinically evaluated qualitatively, is the degree of endpoint trajectory straightness (IC). IC for each direction (ReachIn, ReachOut) correlated with several clinical impairment scores (FMA-UL: *r* = −0.47 *p* < 0.05, *r* = −0.52 *p* < 0.02; FMA-Arm: *r* = −0.52 *p* < 0.02, *r* = −0.69 *p* = 0.001); biceps spasticity: *r* = 0.47 *p* < 0.05, *r* = 0.46 *p* < 0.04; triceps spasticity: *r* = 0.55 *p* < 0.01, *r* = 0.46 *p* < 0.04) and with FNT-time (*r* = 0.76, *p* < 0.001; *r* = 0.91, *p* < 0.001). ReachIn IC also correlated with clinical activity scores (BBT: *r* = −0.57, *p* < 0.01).

### Discriminant validity

**Fig. 5:**
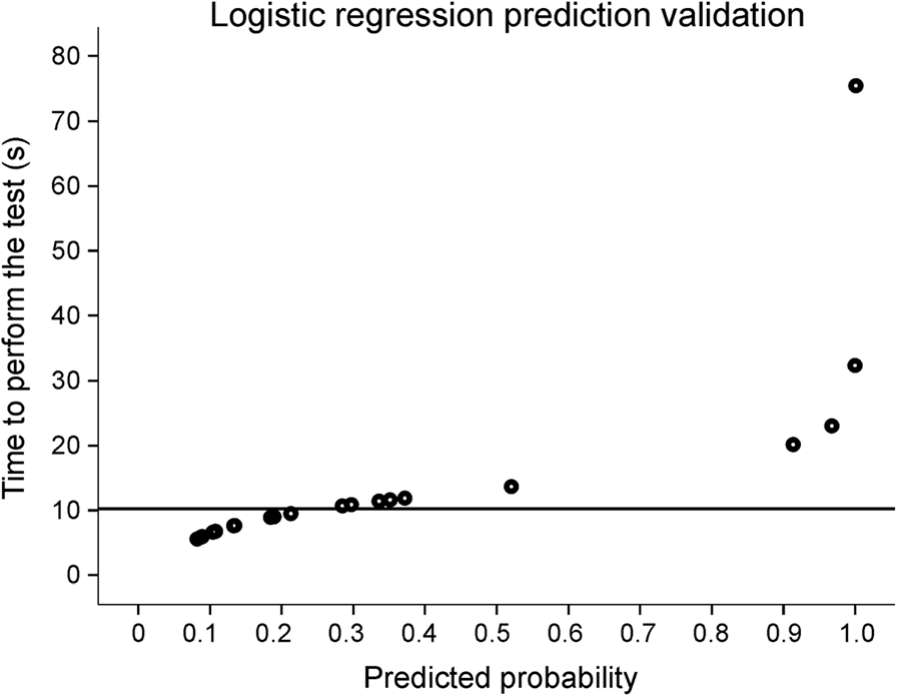
Predicted probabilities plot showing the probability values for each of the patients in the stroke group with an indication of the cut-off time (10.6 s) identified in the logistic regression analysis

The area under the ROC curve indicated excellent discriminatory power of FNT-time (AUC = 0.85, 95% CI = 0.64–1.00), and the Sensitivity/Specificity decision plot identified an optimal cut-off time of 10.6 s with a sensitivity of 0.714 and specificity of 0.692 (Fig. 4c). The Likelihood Ratio test (LR) better fit the data than the intercept-only model (*p* < 0.005). Moreover, the Hosmer and Lemeshow test was not significant (*p* = 0.465), indicating a good fit of the model. The corresponding individual probabilities of the model are shown in Fig. 5. Statistical tests of individual predictors revealed that the likelihood of a having moderate-to-severe impairment was positively related to FNT-time. Specifically, each additional second in FNT-time was associated with a 42% increase in the odds of having moderate-to-severe impairment. The corresponding odds ratio (95% CI) was estimated as 1.42 (0.96; 2.10) with Wald statistics failing to reach statistical significance (*p* = 0.07).

## Discussion

To our knowledge, this is the first study to objectively quantify UL movement patterns and coordination during performance of the FNT between the nose and a sagittal target. We used a single subject position and target placement but the innovation in our approach was the determination of the relationship between FNT-time (metric) and kinematic variables describing endpoint performance, and UL movement quality. This analysis allowed us to examine the construct, convergent and discriminant validity of FNT-time in individuals with stroke. FNT-time was found to be a good measure of interjoint coordination. Compared to movements made at matched speeds in healthy controls, individuals with stroke used less elbow and shoulder joint movement and more trunk displacement for both ReachIn and ReachOut directions. In addition, while the interjoint coordination pattern differed in the healthy subjects according to movement direction, individuals with stroke used a similar spatial interjoint coordination pattern for both directions. Overall, the temporal interjoint coordination score (LAG) was an excellent predictor of the variance in the time to perform FNT and FNT-time was related to clinical impairment.

### Construct validity: relationship between FNT-time and UL coordination

In healthy subjects making slow arm movements, FNT-time was not linked to any particular UL movement pattern, indicating that they could use different combinations of joint rotations to achieve the same hand path (motor equivalence) [30–32]. Of note was that healthy subjects adapted the interjoint coordination pattern in order to perform the test faster by increasing shoulder-elbow temporal coupling. This type of speed-driven movement pattern adaptation is consistent with notions of the control of fast movement [33, 34].

Consistent with previous studies [35, 36], individuals with stroke took longer to perform exocentric (ReachOut) compared to egocentric (ReachIn) movement. The time to perform FNT was influenced by higher shoulder-elbow temporal coupling (LAG) during exocentric compared to egocentric movement. Indeed, movements in each direction were likely influenced by the presence of pathological extensor and flexor UL synergies [10, 37, 38]. The influence of pathological synergies may decrease the variability of UL movement patterns used for functional tasks [31, 35, 36]. Previous studies of UL interjoint coordination in stroke have mainly focused on exocentric movements showing a disruption in the relative timing of shoulder and elbow movements in reaching towards targets in different parts of the arm workspace (e.g., near, far, contralateral, ipsilateral) [39]. Our results suggest that the disruption in temporal interjoint coordination affects movements in both directions, which is well-captured in this version of FNT.

### Convergent validity: FNT-time and clinical outcomes

In patients with ataxia, FNT-time has shown convergent validity with gross and fine finger dexterity (*r* = 0.82), functional independence (*r* = 0.74) and social participation (*r* = 0.78) [40]. FNT-time also discriminates between levels of UL function in healthy older individuals (gross/fine manual dexterity, grip strength), and correlates with Box-and-Blocks (*r* = 0.82), Purdue Pegboard (*r* = 0.82) [17], and functional arm tests (*r* = 0.70–0.84) [41]. We showed that, in subjects with stroke, FNT-time was related to whole limb UL impairment severity (FMA-UL), arm impairment only (FMA-Arm) as well as activity limitations. Although a version of FNT is included in FMA-UL, the nature of movements and the scoring of the FNT portion of the FMA-UL differ from the FNT studied here. Scoring of the FMA-UL version is based on the difference in time between movements of the affected and non-affected arms instead of the actual time to perform the test. The FMA-UL version of the FNT also only accounts for movements performed in an egocentric frame of references (subject’s own nose and knee). Our description of the relationship between FNT-time and impairment severity in patients with stroke is consistent with previous studies in patients with other neurological pathology (head injury [17], multiple sclerosis [41], neuromuscular disorders) [40].

### Discriminant validity

FNT-time differentiated between individuals with mild and moderate-to-severe stroke when using FMA-UL (cut-off = 50/66) and had high discriminative power (AUC = 0.85). Thus, for this version of the Finger-to-Nose test, subjects with mild and moderate-to-severe impairment performed the test faster or slower than 10.6 s, respectively, validating the objective metric of the test (time) to differentiate between levels of severity in individuals with chronic stroke.

### Study limitations

Individuals had stroke-related deficits but inclusion was limited to those who could perform the test (Chedoke-McMaster level 3/7). Results cannot be generalized to people with more severe stroke. Participants in the study could visualize the target and make accurate reaching movements. However, since we did not assess visual or perceptual deficits per se, we cannot generalize the conclusions to patients with these deficits. Conclusions that the metric of time is a good indicator of UL coordination in individuals with stroke is limited to the version of the FNT performed in this analysis.

## Conclusions

Understanding how the damaged nervous system uses its available kinematic redundancy is relevant for both practice and research in rehabilitation. The assessment and quantification of motor redundancy and adaptability is likely to be essential for the measurement of treatment efficacy and recovery leading to improvement in patient care [31]. Our study showed that FNT-time reflected temporal and spatial interjoint coordination, validating the test construct. In addition, dividing the ego- and exocentric movement direction analysis provided insights into clinically relevant direction-dependent movement deficits and their relationship with pathological UL synergies. This is a new approach to understanding the role of synergies during arm movements that include changes in direction. FNT-time was correlated with clinical impairment (FMA-UL, FMA-Arm) and activity limitation (BBT) demonstrating its convergent validity. In addition, FNT-time discriminated between mild and moderate-to-severe impairment levels in individuals with stroke.

Validation of the FNT-time is relevant to both clinicians and researchers interested in the evaluation of UL coordination deficits. Results suggest that FNT-time is a valid assessment of UL coordination and can be used to monitor post-stroke recovery.

## Abbreviations

BBT: Box and Blocks test
CRIR: Centre for Interdisciplinary Research in Rehabilitation of Greater Montreal
FMA: Fugl-Meyer Assessment
FNT: Finger-to-Nose test
IC: Index of curvature
IJC: Spatial interjoint coordination
LAG: Temporal cross-correlation index
ROC: Receiver Operating Characteristic
UL: Upper-limb
